# Maximum Correntropy Criterion Kalman/Allan Variance-Assisted FIR Integrated Filter for Indoor Localization

**DOI:** 10.3390/mi16030303

**Published:** 2025-03-04

**Authors:** Manman Li, Lei Deng, Yide Zhang, Yuan Xu, Yanli Gao

**Affiliations:** 1 School of Electrical Engineering, Shandong Huayu University of Technology, Dezhou 253034, China; lmm@huayu.edu.cn (M.L.);; 2 School of Electrical Engineering, University of Jinan, Jinan 250022, China; 202221200888@stu.ujn.edu.cn (Y.Z.); cse_xuy@ujn.edu.cn (Y.X.); 3 Jinan Chenhe Information Technology Co., Ltd., Jinan 250022, China; 4 Qingdao Branch of Naval Aviation University, Qingdao 266000, China; 5 Faculty of Information Science and Engineering, Ocean University of China, Qingdao 266000, China

**Keywords:** maximum correntropy criterion, Kalman filter, finite impulse response (FIR), inertial navigation system (INS)

## Abstract

To obtain more accurate information on using an inertial navigation system (INS)-based integrated localization system, an integrated filter with maximum correntropy criterion Kalman filter (mccKF) and finite impulse response (FIR) is proposed for the fusion of INS-based multisource sensor data in this work. In the realm of medical applications, precise localization is crucial for various aspects, such as tracking the movement of a medical instrument within the human body or monitoring its position in the human body during procedures. This study uses ultra-wideband (UWB) technology to rectify the position errors of the INS. In this method, the difference between the positions of the INS and UWB is used as the measurement of the filter. The main data fusion filter in this study is the mccKF, which utilizes the maximum correntropy criterion (mcc) method to enhance the robustness of the Kalman filter (KF). This filter is used for fusing data from multiple sources, including the INS. Moreover, we use the *Mahalanobis* distance to verify the performance of the mccKF. If the performance of the mccKF is lower than the preset threshold, the Allan Variance-assisted FIR filter is used to replace the mccKF, which is designed in this work. This adaptive approach ensures the resilience of the system in demanding medical environments. Two practical experiments were performed to evaluate the effectiveness of the proposed approach. The findings indicate that the mccKF/FIR integrated method reduces the localization error by approximately 32.43% and 37.5% compared with the KF and mccKF, respectively. These results highlight the effectiveness of the proposed approach.

## 1. Introduction

Nowadays, the inertial navigation system (INS)-based measurement is widely used in several fields [1]. For instance, in the research performed by [2], a design integrating multisensor fusion was suggested. This design includes an INS, a global navigation satellite system (GNSS), and light detection and ranging (LiDAR) for implementing 3D simultaneous localization and mapping (SLAM; INS/GNSS/3D LiDAR-SLAM). Furthermore, the research performed in [3] delved into the utilization of GNSSs/INSs for assisted imaging systems in uncrewed aerial vehicles (UAVs). In addition, a method for analyzing gait in individuals with Parkinson’s disease using wearable technology based on the INS was designed to evaluate motor ability in [4]. Notably, INS-based measurement technology is gradually becoming a research hotspot.

In INS-based localization, reducing the position cumulative error of the INS is the most important issue that needs to be addressed [5]. Several approaches have been proposed to overcome this problem based on two aspects: integrated navigation methods and data fusion filters. The integrated navigation method has several approaches. For instance, in [6], a GNSS/INS integrated navigation system is supported by a back propagation (BP) neural network with motion constraints. This approach offers supplementary information to address the issue of rapid divergence in the positioning error of integrated navigation systems during GNSS outages. A new robust adaptive method, utilizing a tight real-time kinematic (RTK)/INS architecture, was proposed in [7]. It enhances the robust estimation method by considering two factors, including ambiguity variance, for RTK-related observations. To overcome the problem of inaccuracy in lateral velocity constraint parameters, a navigation method based on a BP neural network for an integrated GNSS/INS/odometer (OD) with nonholonomic constraints (NHCs) has been proposed [8], considering the state of vehicle motion. In the work by the authors of [9], the utilization of an adaptive model set aims to increase the effectiveness of an integrated navigation system compared to an INS with Doppler velocity log in challenging measurement environments. In [10], a microelectromechanical system (MEMS)–INS-based ultra-wideband (UWB) integrated method for indoor navigation was designed. Notably, the principle of the abovementioned examples is to employ different navigation and positioning technologies to continuously correct the calculation errors in INS navigation.

Based on the INS integrated navigation scheme, a data fusion filter can further improve the localization accuracy [11]. Meanwhile, several approaches have been proposed to deal with the data fusion problem. For example, in [12], one robust Kalman filter (KF) was proposed for loosely coupled global positioning system (GPS)/INS integration systems. A dual-rate KF has been devised for integrating time-differenced GPS measurements with INS data [13]. For nonlinear cases, the extended KF (EKF) was considered in [14]. In [15], the EKF was proposed for INS/GPS integrated navigation. Meanwhile, a locally weighted linear regression (LWLR)/long short-term memory (LSTM)-assisted EKF was designed for the integrated navigation of an INS/celestial navigation system [16]. Notably, Kalman-based approaches for data fusion have difficulty in overcoming the disadvantage of accurately capturing environmental noise. To overcome this problem, the finite impulse response (FIR) filter was developed in [17]. The FIR filter only uses the size of the data fusion window and state vector. Alternatively, the FIR filter only requires the size of the data fusion window because the data fusion model is already built. Simultaneously, several approaches have been suggested. For example, in [18], the extended FIR filter was introduced for the fusion of recent INS and UWB measurements. One self-tuning FIR filter has been proposed for processes with unknown measurement noise covariance [19]. Notably, the calculation of the size of the optimal data fusion window is considerably important.

Nowadays, both the mccKF and the FIR have been widely used in localization. However, is should be pointed out that there are many shortcomings. For mccKF, this method can improve the robustness; however, the matrix in the estimation process is prone to singularity, which can cause the mccKF to not work. Moreover, the accuracy of the noise will also affect the localization accuracy. The FIR filter has better robustness; however, if the noise of the filter affects the accuracy, we have to say that its performance is worse than that of the KF method. Thus, we propose an mccKF/FIR filtering approach; when the KF method cannot provide a stable solution, the FIR can. To obtain more accurate position information using the INS-based integrated localization system, a maximum correntropy criterion KF (mccKF)/FIR) integrated filter is proposed for the fusion of INS-based multisource sensor data in this work. Herein, UWB is used to correct the position error of the INS. In this method, the difference between the positions of the INS and UWB is used as the measurement of the filter. The main data fusion filter in this context is mccKF, which uses the mcc method to improve the robustness of the KF. Its application involves the fusion of INS-based multisource sensory data. Moreover, we use the *Mahalanobis* distance to verify the performance of the mccKF. If the performance of the mccKF is below the preset threshold, an Allan Variance-assisted FIR filter is used to replace the mccKF, which employs the Allan Variance method to improve the noise accuracy. Two practical tests were performed to evaluate the effectiveness of the proposed methodology. The results of these experiments verify the efficacy of the proposed strategy.

The remaining sections of this paper are organized as follows: Section 2 discusses the principle of indoor integrated localization system by fusing the INS and UWB. Section 3 presents the design of an mccKF/FIR integrated filter for INS-based integrated navigation. The analysis of the experimental tests is summarized in Section 4, and the conclusions are provided in Section 5.

## 2. Indoor Integrated Localization by Fusing INS and UWB

This subsection discusses the design of the indoor integrated localization scheme by combining INS and UWB data. Furthermore, we briefly introduce the state and measurement equations used by the proposed filter.

### 2.1. Indoor Integrated Localization Scheme

**Figure 1:**
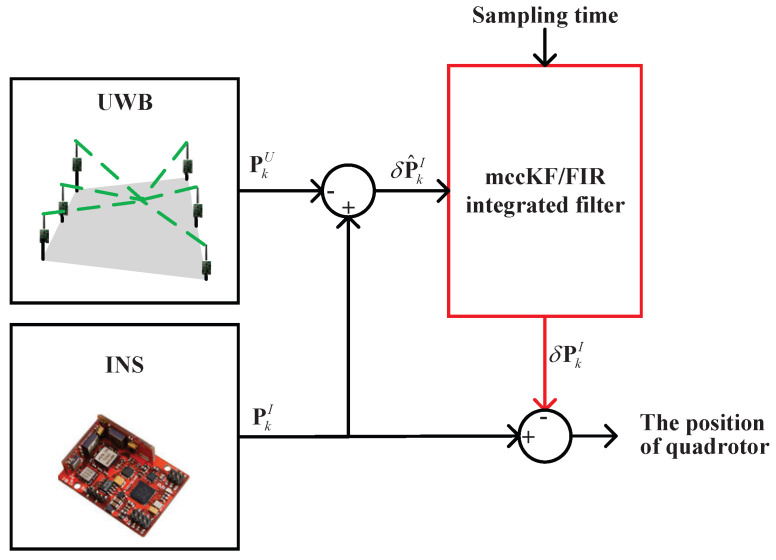
Indoor integrated localization scheme obtained by fusing INS and UWB data.

Figure 1 illustrates the indoor integrated localization scheme, which combines INS and UWB data. The figure shows that the integrated localization scheme uses UWB and the INS to obtain the position of UWB- and INS-based target drones $P_{k}^{UWB}$ and $P_{k}^{INS}$ at time index *k*, respectively. Subsequently, the difference $\delta {\hat{P}}_{k}^{INS}=P_{k}^{INS}-P_{k}^{UWB}$ between $P_{k}^{UWB}$ and $P_{k}^{INS}$ is used as the measurement of the mccKF/FIR integrated filter, which will be discussed in detail in the subsequent section. Meanwhile, the sampling time of the integrated localization scheme is used as the measurement of the mccKF/FIR integrated filter. With these measurements, the mccKF/FIR integrated filter outputs the estimation of the position $\delta P_{k}^{INS}$, and this value is used to correct the $P_{k}^{INS}$.

### 2.2. State and Measurement Equations

Expanding on the integrated scheme described in Section 2.1, the state and measurement equations of the mccKF/FIR integrated filter are presented. The state equation used herein is presented as Equation (1).

(1) $$\underset{\delta s_{k}^{-}}{\underset{︸}{\left[\begin{array}{c} \delta s_{E,k}^{-}\\ \delta s_{N,k}^{-}\\ \delta s_{U,k}^{-}\\ \Delta k_{k}^{-} \end{array}\right]}}=\underset{F}{\underset{︸}{\left[\begin{array}{cccc} F_{E} & 0 & 0 & 0\\ 0 & F_{N} & 0 & 0\\ 0 & 0 & F_{U} & 0\\ 0 & 0 & 0 & 1 \end{array}\right]}}\underset{\delta s_{k-1}}{\underset{︸}{\left[\begin{array}{c} \delta s_{E,k-1}\\ \delta s_{N,k-1}\\ \delta s_{U,k-1}\\ \Delta k_{k} \end{array}\right]}}+\varpi_{k}\;,$$

where $\delta s_{E,k}^{-}={\left[\begin{array}{cc} \delta P_{E,k}^{-} & \delta V_{E,k}^{-} \end{array}\right]}^{T}$, $\delta s_{N,k}^{-}={\left[\begin{array}{cc} \delta P_{N,k}^{-} & \delta V_{N,k}^{-} \end{array}\right]}^{T}$, $\delta s_{U,k}^{-}={\left[\begin{array}{cc} \delta P_{U,k}^{-} & \delta V_{U,k}^{-} \end{array}\right]}^{T}$, $\delta s_{E,k}={\left[\begin{array}{cc} \delta P_{E,k}^{-} & \delta V_{E,k}^{-} \end{array}\right]}^{T}$, $\delta s_{N,k}={\left[\begin{array}{cc} \delta P_{N,k} & \delta V_{N,k} \end{array}\right]}^{T}$, $\delta s_{U,k}={\left[\begin{array}{cc} \delta P_{U,k} & \delta V_{U,k} \end{array}\right]}^{T}$, and $\varpi_{k}∼N\left(0,Q_{k}\right)$ represents the system noise, and $F_{E}$, $F_{N}$, and $F_{U}$ can be defined as follows:

(2) $$F_{E}=F_{N}=F_{U}=\left[\begin{array}{cc} 1 & \Delta k\\ 0 & 1 \end{array}\right]\;,$$

where Δk represents the sampling time. The measurement equation utilized herein can be expressed as follows:

(3) $$y_{k}=\left[\begin{array}{c} P_{E,k}^{I}-P_{E,k}^{U}\\ P_{N,k}^{I}-P_{N,k}^{U}\\ P_{U,k}^{I}-P_{U,k}^{U}\\ \Delta \hat{k} \end{array}\right]=\underset{H}{\underset{︸}{\left[\begin{array}{ccccccc} 1 & 0 & 0 & 0 & 0 & 0 & 0\\ 0 & 0 & 1 & 0 & 0 & 0 & 0\\ 0 & 0 & 0 & 0 & 1 & 0 & 0\\ 0 & 0 & 0 & 0 & 0 & 0 & 1 \end{array}\right]}}\delta s_{k}^{-}+\upsilon_{k}\;,$$

where $\left(P_{E,k}^{INS},P_{N,k}^{INS},P_{U,k}^{INS}\right)$ represents the position of the INS-based target drone at time index *k*, ${\left(P_{E,k}^{UWB},P_{N,k}^{UWB},P_{U,k}\right)}^{UWB}$ represents the position of the UWB-based target drone at time index *k*, and $\upsilon_{k}∼N\left(0,R_{k}\right)$ represents the measurement noise.

## 3. mccKF/FIR Integrated Filter

In this section, the mccKF/FIR integrated filter mentioned in Section 2.1 is presented. First, we provide a brief introduction to the mccKF method. Subsequently, the FIR filter based on the state and measurement equations mentioned in Section 2.2 is constructed. Finally, the mccKF/FIR integrated filter is proposed.

### 3.1. Maximum Correntropy Criterion

Correntropy quantifies the resemblance between two random variables A,B∈R:

(4) $$C\left(A,B\right)=E\left[\alpha \left(A,B\right)\right]=\int {\alpha \left(A,B\right)df}_{AB}\left(A,B\right)\;,$$

where $f_{AB}\left(A,B\right)$ represents the joint distribution function. And $\alpha \left(A,B\right)$ represents its kernel, which is defined as follows:

(5) $$\alpha \left(A,B\right)=G_{\sigma }\left(A-B\right)=\exp\left(-\frac{{\left(A-B\right)}^{2}}{\sigma }\right)\;,$$

Notably, obtaining $f_{AB}\left(A,B\right)$ is challenging. Therefore, we can estimate the correntropy using the following equation:

(6) $$\hat{C}\left(A,B\right)=\frac{1}{M}\sum_{g=1}^{M}G_{\sigma }\left(\delta e\left(g\right)\right)\;,$$

where $\delta e\left(g\right)=A\left(g\right)-B\left(g\right)$. In this work, we set $\delta e\left(g\right),g\in \left[1,M\right]$ is available, and the mcc cost function can be written as follows:

(7) $$\max J=\max\frac{1}{M}\sum_{g=1}^{M}G_{\sigma }\left(\delta e\left(g\right)\right)\;,$$

### 3.2. mccKF Filter

With model (1) and (2), the data fusion filter, with the KF being the most famous example, has the potential to enhance localization accuracy. Based on model (1) and (2), the KF method initially uses one-step prediction, which can be written as follows:

(8) $$\delta s_{k}^{KF-}=F\delta s_{k-1}^{KF}+{\varpi_{k}}^{KF}\;,$$

(9) $$P_{k}^{KF-}=FP_{k-1}^{KF}F^{T}+Q\;,$$

Subsequently, with measurement $y_{k}$, the KF uses the updated measurement to provide the estimated value using the following equations:

(10) $$K_{k}^{KF}=P_{k}^{KF-}H^{T}{\left(HP_{k}^{KF-}H^{T}+R\right)}^{-1}\;,$$

(11) $$\delta s_{k}^{KF}=\delta s_{k}^{KF-}+K_{k}^{KF}\left(y_{k}-H\delta s_{k}^{KF-}\right)\;,$$

(12) $$P_{k}^{KF}=\left(I-K_{k}^{KF}H\right)P_{k}^{KF-}\;,$$

The KF algorithm based on model (1) and (2) can be listed as Algorithm 1.
**Algorithm 1:** KF algorithm based on model (1) and (2)
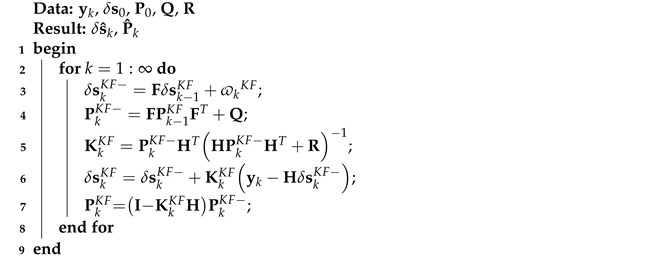


The mcc-KF employs the mcc as the optimization criterion to obtain the posterior estimates, which is robust against heavy-tailed non-Gaussian noises. We rewrite Equations (1) and (2) using Equations (8) and (9) as follows:

(13) $$\left[\begin{array}{c} \delta {\hat{s}}_{k}\\ y_{k} \end{array}\right]=\left[\begin{array}{c} I\\ H \end{array}\right]\delta s_{k}+\Lambda_{k}\;,$$

where

(14) $$\Lambda_{k}=\left[\begin{array}{c} -\left(\delta s_{k}-\delta {\hat{s}}_{k}\right)\\ \upsilon_{k} \end{array}\right]\;,$$

And we obtain the following equation:

(15) $$E\left[\Lambda_{k}\Lambda_{k}^{T}\right]=\left[\begin{array}{cc} P_{k}^{KF} & 0\\ 0 & R_{k} \end{array}\right]=\left[\begin{array}{cc} \chi_{k,P_{k}^{KF}}^{KF}{\left(\chi_{k,P_{k}^{KF}}^{KF}\right)}^{T} & 0\\ 0 & \chi_{k,R_{k}}^{KF}{\left(\chi_{k,R_{k}}^{KF}\right)}^{T} \end{array}\right]=\chi_{k}^{KF}{\left(\chi_{k}^{KF}\right)}^{T}\;,$$

where $\chi_{k}^{KF}$ can be computed using Cholesky decomposition. In addition, Equation (13) can be rewritten as follows:

(16) $$\underset{y_{k}^{1}}{\underset{︸}{{\left(\chi_{k}^{KF}\right)}^{-1}\left[\begin{array}{c} \delta {\hat{s}}_{k}\\ y_{k} \end{array}\right]}}=\underset{H_{k}^{1}}{\underset{︸}{{\left(\chi_{k}^{KF}\right)}^{-1}\left[\begin{array}{c} I\\ H \end{array}\right]}}\delta s_{k}+\underset{\upsilon_{k}^{1}}{\underset{︸}{{\left(\chi_{k}^{KF}\right)}^{-1}\Lambda_{k}}}\;,$$

Thus, the optimal estimate of $\delta {\hat{s}}_{k}$ can be computed as follows:

(17) $$\delta {\hat{s}}_{k}^{KF}=\underset{\delta s_{k}^{KF}}{\arg\max}J\left(\delta s_{k}^{KF}\right)=\underset{\delta s_{k}^{KF}}{\arg\max}\sum_{g=1}^{M}G_{\sigma }\left(\delta e\left(g\right)\right)\;,$$

Consequently, we can obtain one fixed-point iterative algorithm, and the steps of this method can be expressed as follows:

(18) $$G_{\delta {\hat{s}}_{k}^{KF}}^{KF\left(s-1\right)}=diag\left(G_{\sigma }\left(\delta e_{1,k}^{\left(s-1\right)}\right),G_{\sigma }\left(\delta e_{2,k}^{\left(s-1\right)}\right),...,G_{\sigma }\left(\delta e_{7,k}^{\left(s-1\right)}\right)\right)\;,$$

(19) $$G_{y_{k}}^{KF\left(s-1\right)}=diag\left(G_{\sigma }\left(\delta e_{8,k}^{\left(s-1\right)}\right),G_{\sigma }\left(\delta e_{9,k}^{\left(s-1\right)}\right),G_{\sigma }\left(\delta e_{10,k}^{\left(s-1\right)}\right),G_{\sigma }\left(\delta e_{11,k}^{\left(s-1\right)}\right)\right)\;,$$

(20) $${\hat{P}}_{k}^{KF\left(s-1\right)}=\chi_{k,P_{k}^{KF}}^{KF}{\left(G_{\delta {\hat{s}}_{k}^{KF}}^{KF\left(s-1\right)}\right)}^{-1}{\left(\chi_{k,P_{k}^{KF}}^{KF}\right)}^{T}\;,$$

(21) $$R_{k}^{KF\left(s-1\right)}=\chi_{k,R_{k}}^{KF}{\left(G_{y_{k}}^{KF\left(s-1\right)}\right)}^{-1}{\left(\chi_{k,R_{k}}^{KF}\right)}^{T}\;,$$

(22) $$\delta e\left(g\right)=y_{k,g}^{1}-H_{k,g}^{1}\delta s_{k,g}\;,$$

(23) $$K_{k}^{KF\left(s-1\right)}={\hat{P}}_{k}^{KF\left(s-1\right)}H^{T}{\left({H\hat{P}}_{k}^{KF\left(s-1\right)}H^{T}+R_{k}^{KF\left(s-1\right)}\right)}^{-1}\;,$$

(24) $$\delta {\hat{s}}_{k}^{KF\left(s\right)}=\delta s_{k}^{KF-}+K_{k}^{KF\left(s-1\right)}\left(y_{k}-H\delta s_{k}^{KF-}\right)\;,$$

Thus, the mccKF based on model (1) and (2) can be listed in Algorithm 2.
**Algorithm 2:** The mccKF algorithm based on model (1) and (2)
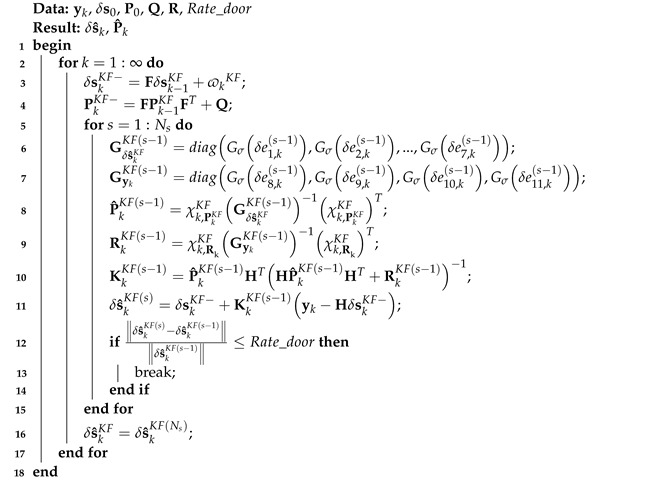


### 3.3. Allan Variance

In this work, we employ Allan Variance to assist the FIR filter, which is able to improve the accuracy of the noise estimation. Firstly, we are able to obtain the following equation with $y_{k}$:

(25) $$\sigma_{y_{k}}^{2}=\frac{1}{2}E\left({\left(y_{k+1}-y_{k}\right)}^{2}\right)\;$$

Based on Equation (25), we can obtain that

(26) $${\hat{R}}_{k}=\frac{1}{2\left(k-1\right)}\sum_{j=1}^{N-1}\left[{\left(y_{k+1}-y_{k}\right)}^{2}\right]\;,$$

where *N* is the sum of the data groups. Then, we can obtain

(27) $${\hat{R}}_{k}=\left(1-\lambda_{k-1}\right){\hat{R}}_{k-1}+\frac{1}{2}\lambda_{k-1}{\left(y_{k}-y_{k-1}\right)}^{2}\;,$$

### 3.4. FIR Filter

It is evident from the aforementioned section that the mccKF filter enhances robustness compared with the conventional KF method. However, the mccKF depends on noise statistics. Meanwhile, the FIR filter maintains the robustness of the proposed filter. Based on model (1) and (2), the FIR filter estimates the error of the INS using the measurement at time index $j\in [k-N_{F}+1,k]$. The FIR filter uses the KF to fuse the data in the dead zone. When the time index is $k>N_{F}$, where $N_{F}$ is the size of the filtering window, the FIR performs one-step prediction, which is listed as follows:

(28) $$\delta s_{j}^{F-}=F\delta s_{j}^{F}\;,$$

(29) $$P_{j}^{F-}={\mathrm{FP}}_{j}^{F}F^{T}+Q\;,$$

Then, the measurement update can be performed using the following equations:

(30) $$G_{v}^{F}={\left(H^{T}H+{\left(\mathrm{FH}F^{T}\right)}^{-1}\right)}^{-1}\;,$$

(31) $$K_{v}^{F}=G_{v}^{F}H\;,$$

(32) $$\delta {\bar{s}}_{v}^{F}=\delta {\bar{s}}_{v}^{F-}+K_{v}^{F}\left(y_{v}-H\delta {\bar{s}}_{v}^{F-}\right)\;,$$

(33) $${\bar{P}}_{v}^{F}=\left(I-K_{v}^{F}H\right){\bar{P}}_{v}^{F-}\;,$$

Thus, the FIR filter based on model (1) and (2) can be listed in Algorithm 3.
**Algorithm 3:** FIR filter algorithm based on model (1) and (2)
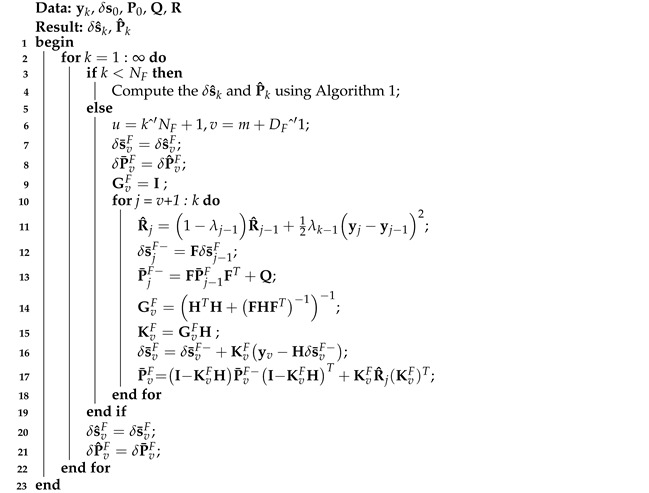


### 3.5. mccKF/FIR Integrated Filter

**Figure 2:**
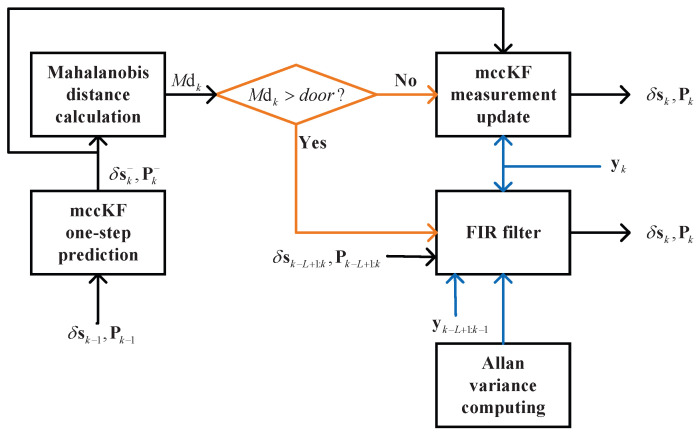
Scheme of the mccKF/FIR integrated filter.

In this study, we used the mccKF/FIR integrated filter, which is shown in Figure 2. First, we performed the one-step prediction of the mccKF $\delta s_{k}^{-}$ and $P_{k}^{-}$ with $\delta s_{k-1}$ and $P_{k-1}$. The Mahalanobis distance can be used to determine differences in the data distribution. Subsequently, the Mahalanobis distance is computed using the following equation:

(34) $$Md_{k}={\left(y_{k}-H\delta s_{k}^{KF-}\right)}^{T}R\left(y_{k}-H\delta s_{k}^{KF-}\right)\;,$$

When $Md_{k}>door$, we used the FIR filter to provide the output, and when $Md_{k}<door$, we directly updated the measurement.

## 4. Results

In this study, a practical test was conducted to validate the efficacy of the proposed approach. First, we provide an overview of the test setup. Second, the localization accuracy of the proposed mccKF/FIR integrated filter is compared with that of a traditional filter.

### 4.1. The Design of the Testbed

**Figure 3:**
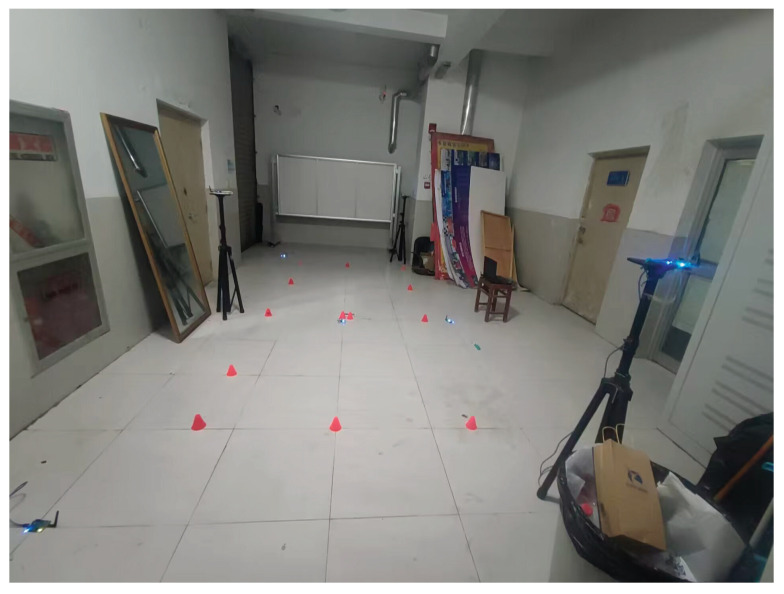
Test environment used in this work.

**Figure 4:**
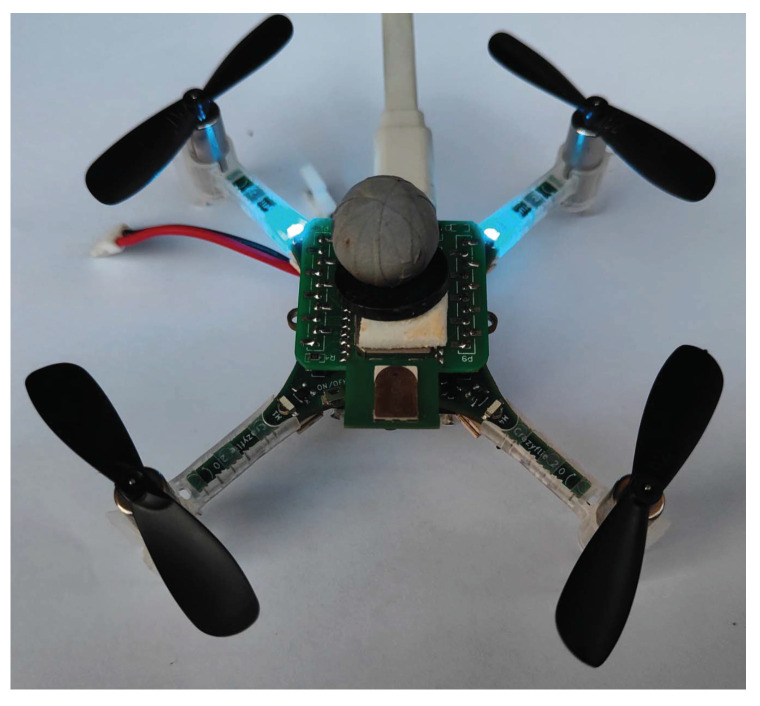
crazyflie2.0 used in the test.

In this subsection, the testbed used herein is presented. In this study, a practical test was conducted in building No. 1 of the University of Jinan, China. Figure 3 and Figure 4 illustrates the real test environment utilized for the experiment. In the test, we used crazyflie2.0 as the target drone, and six DW-1000-based UWB reference nodes (UWB RNs) with known coordinates and one UWB blind node fixed on the target drone to measure $P_{k}^{UWB}$. Meanwhile, one IMU fixed on the target drone was used to measure the INS-derived position $P_{k}^{UWB}$.

#### 4.1.1. Test 1

Herein, two practical tests were performed to demonstrate the enhancements achieved by the proposed method. In this subsection, the result of Test 1 is discussed. First, for the filter, we set Δk=0.02s, $N_{F}=8$, $N_{s}=5$, and Rate_door=0.2, and then $Q_{k}$ and $R_{k}$ were set as follows:

(35) $$Q_{k}=\left[\begin{array}{cc} Q_{1,k} & 0_{4\times 3}\\ 0_{2\times 4} & Q_{2,k}\\ 0_{1\times 4} & Q_{3,k} \end{array}\right]\times 0.25^{2}\;,$$

(36) $$Q_{1,k}=0.01\times \left[\begin{array}{cccc} 0.25\times \Delta k^{2} & 0.5\times \Delta k & 0 & 0\\ 0.5\times \Delta k & 1 & 0 & 0\\ 0 & 0 & 0.25\times \Delta k^{2} & 0.5\times \Delta k\\ 0 & 0 & 0.5\times \Delta k & 1 \end{array}\right]\;,$$

(37) $$Q_{2,k}=\left[\begin{array}{cc} 0.25\times \Delta k^{2} & 0.5\times \Delta k\\ 0.5\times \Delta k & 1 \end{array}\right]\times 1.5^{2}\;,$$

(38) $$R_{k}=\left[\begin{array}{cccc} 0.35 & 0 & 0 & 0\\ 0 & 0.35 & 0 & 0\\ 0 & 0 & 0.05 & 0\\ 0 & 0 & 0 & 0.01 \end{array}\right]\;,$$

(39) $$Q_{3,k}=1\;,$$

**Figure 5:**
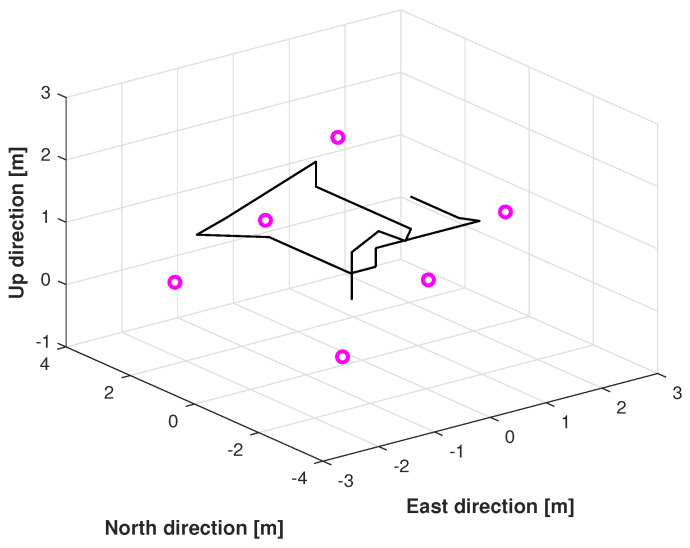
Reference path used in Test 1.

**Figure 6:**
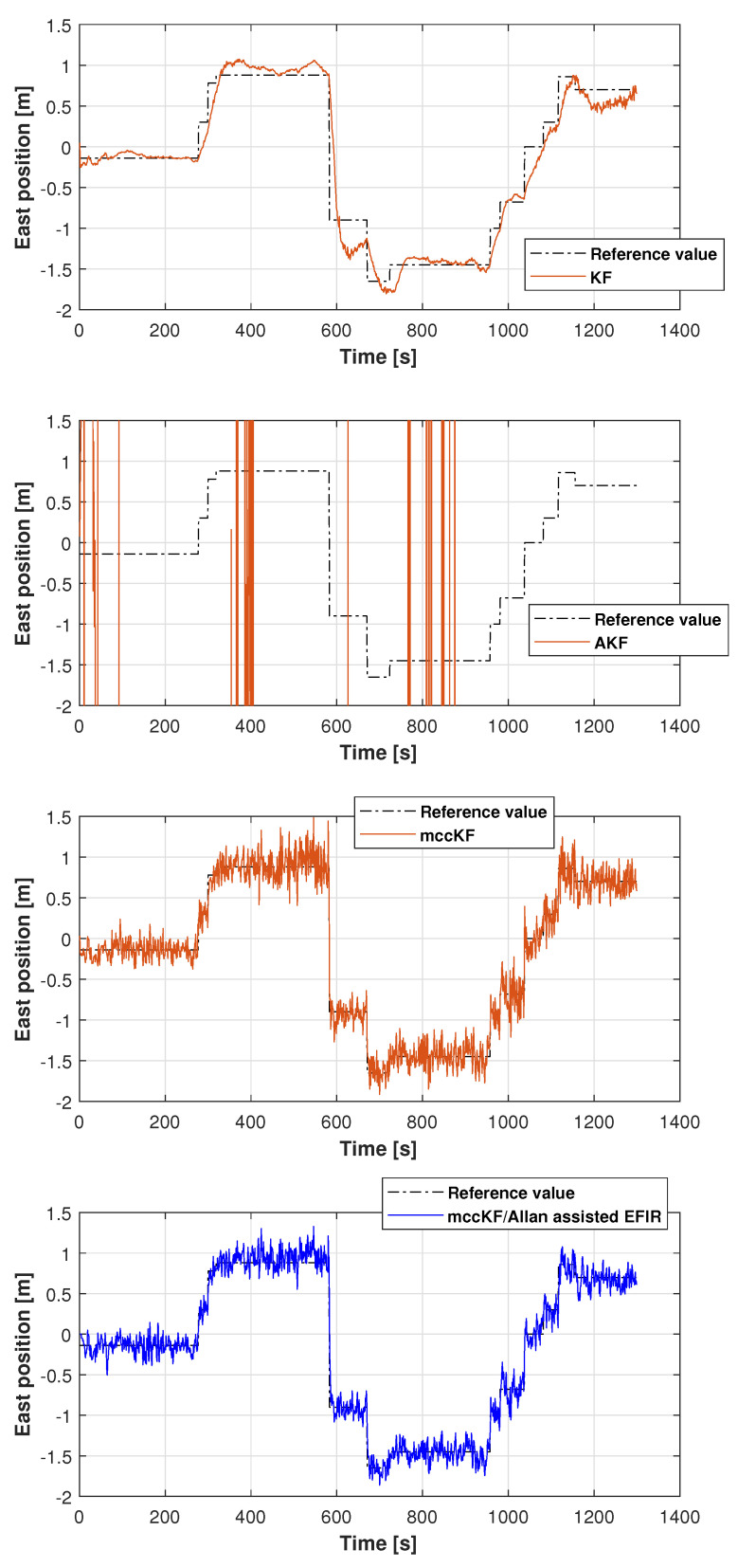
East position measured by the KF, mccKF, and mccKF/Allan-assisted FIR integrated filter in Test 1.

**Figure 7:**
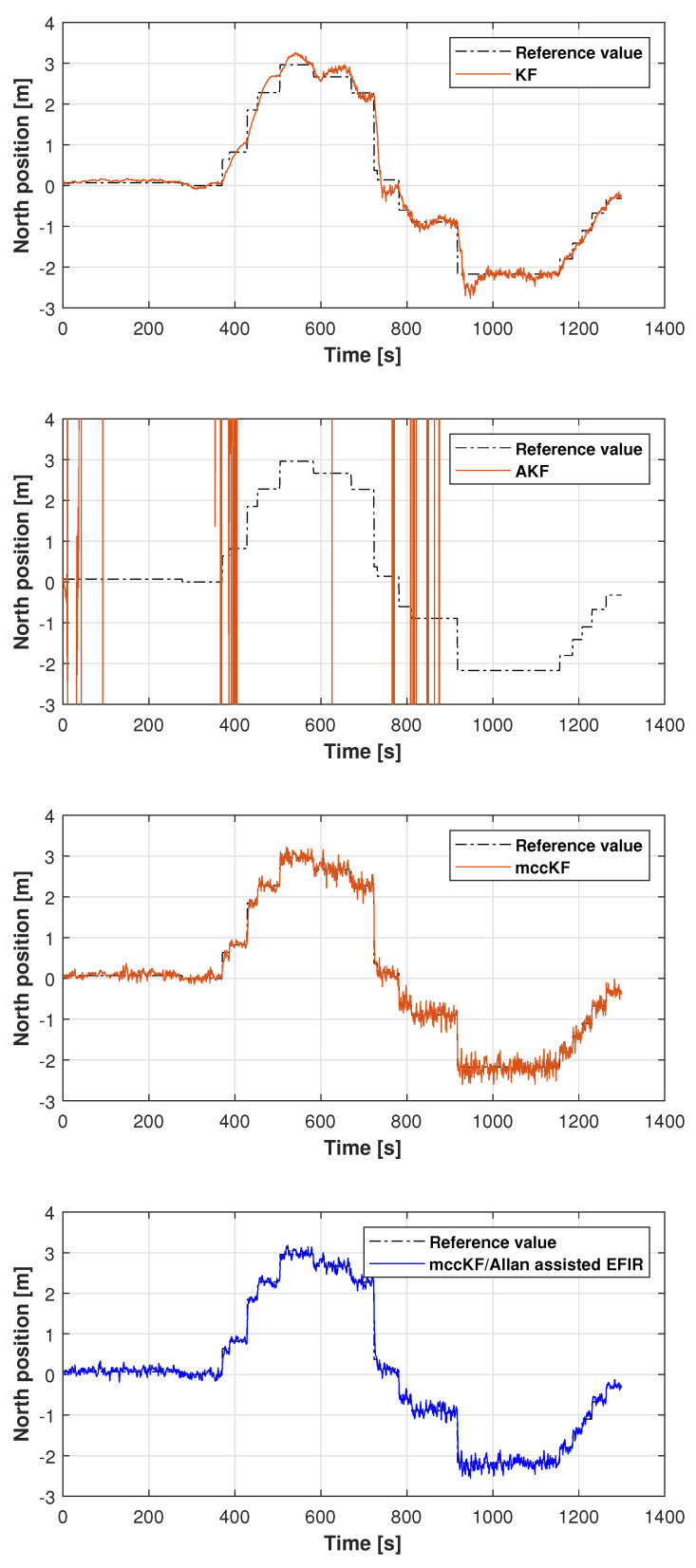
North position measured by the KF, AKF, mccKF, and mccKF/FIR integrated filter in Test 1.

**Figure 8:**
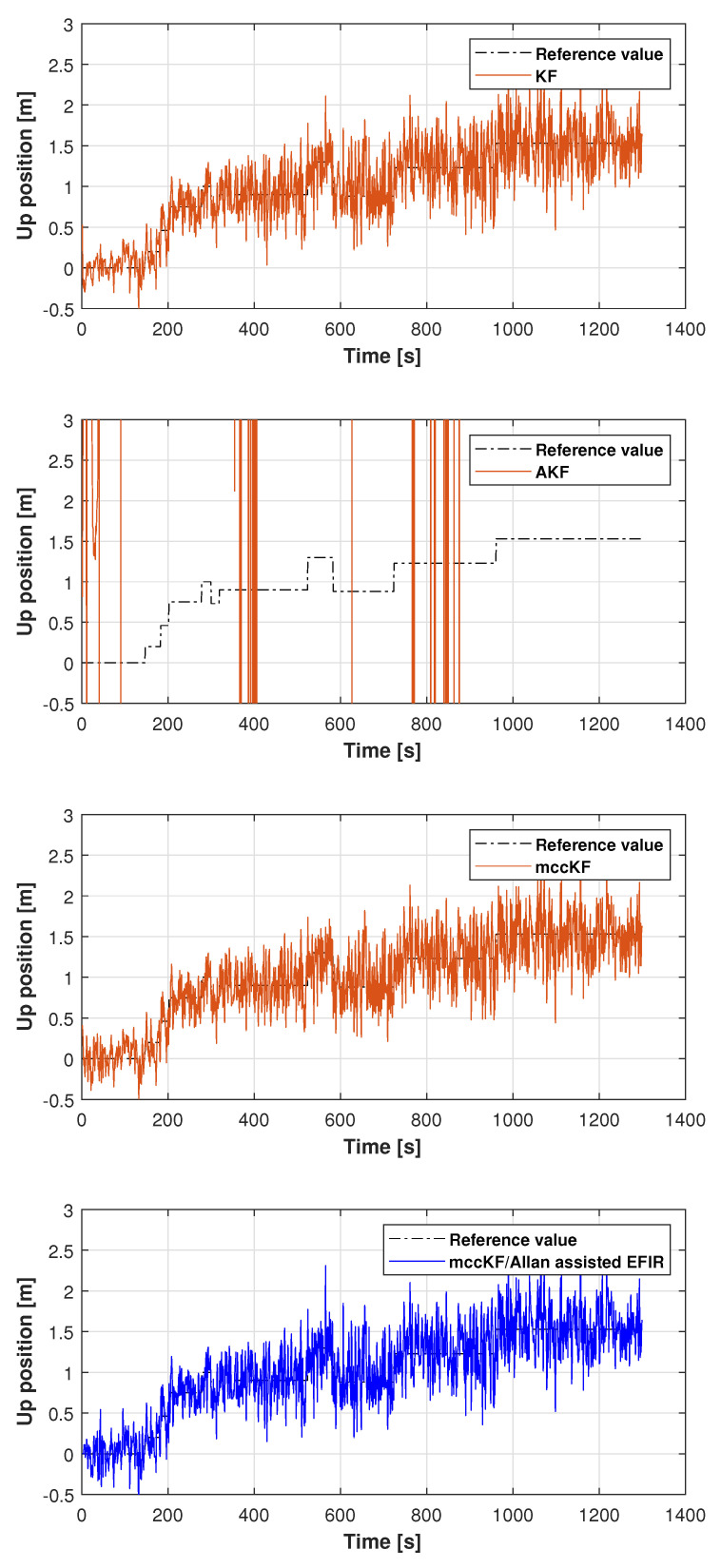
Upward position measured by the KF, AKF, mccKF, and mccKF/Allan-assisted FIR integrated filter in Test 1.

The reference path used in Test 1 is shown in Figure 5, and the positions measured by the KF, adaptive KF (AKF), mccKF, and mccKF/Allan-assisted FIR integrated filters in the east, north, and upward directions in Test 1 are compared in Figure 6, Figure 7 and Figure 8. The figures reveal that the KF, mccKF, and proposed mccKF/Allan-assisted FIR integrated filters provide the position in the east, north, and upward directions, respectively. The AKF has big errors. The situations in Figure 7 and Figure 8 are similar. These figures show that the KF and mccKF exhibit weak divergence in the east and north directions. When compared with the KF and mccKF, the positions measured by the proposed mccKF/FIR integrated filter are closer to the reference position, indicating that the suggested approach is more effective in minimizing localization errors. Figure 8 shows that all solutions of the filters are similar, and the positions measured by the proposed have small fluctuations when compared with other filters.

**Figure 9:**
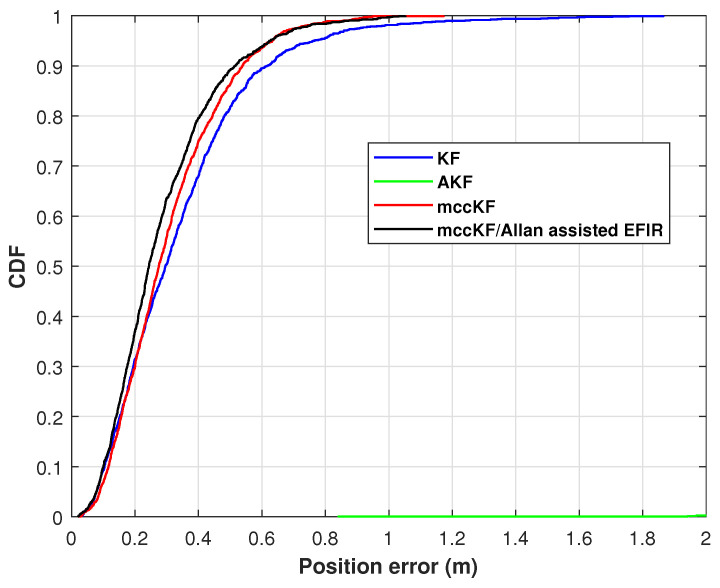
CDF of the position errors measured by the KF, AKF, mccKF, and Allan-assisted mccKF/FIR integrated filter in Test 1.

The cumulative distribution function (CDF) of the position errors, as measured by the KF, AKF, mccKF, and mccKF/Allan-assisted FIR integrated filter in Test 1, are depicted in Figure 9. The figure shows that the localization error of the mccKF is less than that of the KF when the CDF is 0.9. Compared with the mccKF and KF, the proposed mccKF/FIR integrated filter exhibits minimal localization error when the CDF is 0.9. This highlights the efficacy of the suggested approach in minimizing localization errors.

**Figure 10:**
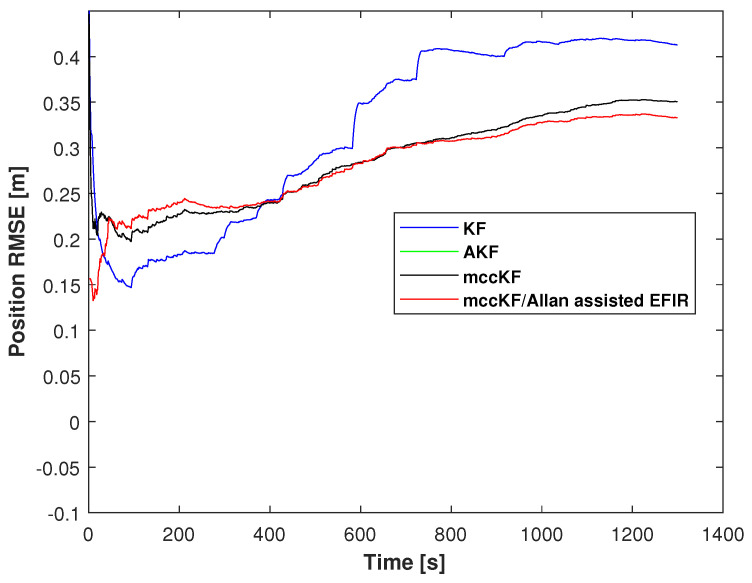
RMSE of the position errors measured by the KF, AKF, mccKF, and Allan-assisted mccKF/FIR integrated filter in Test 1.

To provide a more detailed demonstration of the effectiveness of the proposed approach, we use the position root mean square error (RMSE) in each time index, which is computed using the following equation:

(40) $$RMSE_{k}=\frac{1}{k}\sum_{j=1}^{k}\sqrt{{\left({\hat{P}}_{E,j}-P_{E,j}\right)}^{2}+{\left({\hat{P}}_{N,j}-P_{N,j}\right)}^{2}+{\left({\hat{P}}_{U,j}-P_{U,j}\right)}^{2}}\;,$$

where $\left({\hat{P}}_{E,j},{\hat{P}}_{N,j},{\hat{P}}_{U,j}\right)$ represents the measurement of $\left(P_{E,j},P_{N,j},P_{U,j}\right)$ at time index *j*. And Figure 10 shows the RMSE of the position errors measured by the KF, mccKF, and Allan-assisted mccKF/FIR integrated filter in Test 1. From this figure, we can see that the proposed method has the smallest RMSE errors.

Table 1 lists the position RMSEs of the KF, mccKF, and mccKF/Allan-assisted FIR integrated filter in Test 1. The table shows that the mean RMSE of the mccKF is slightly lower than that of the KF. The mccKF/Allan-assisted FIR integrated filter reduces the localization error from 0.23 to 0.16 m when compared with the KF, which has the lowest localization error. All the figures and the table illustrate that, in Test 1, the proposed mccKF/Allan-assisted FIR filter is more efficient in minimizing localization errors when compared with the KF and mccKF.

#### 4.1.2. Test 2

**Figure 11:**
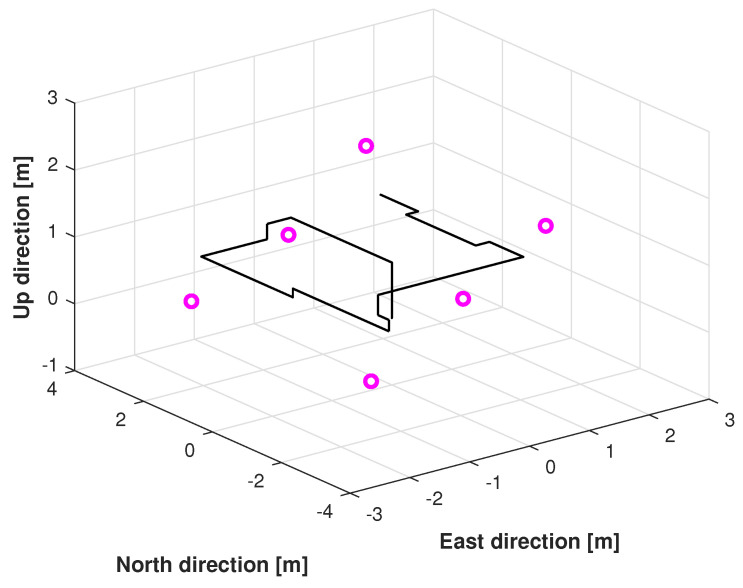
Reference path used in Test 2.

**Figure 12:**
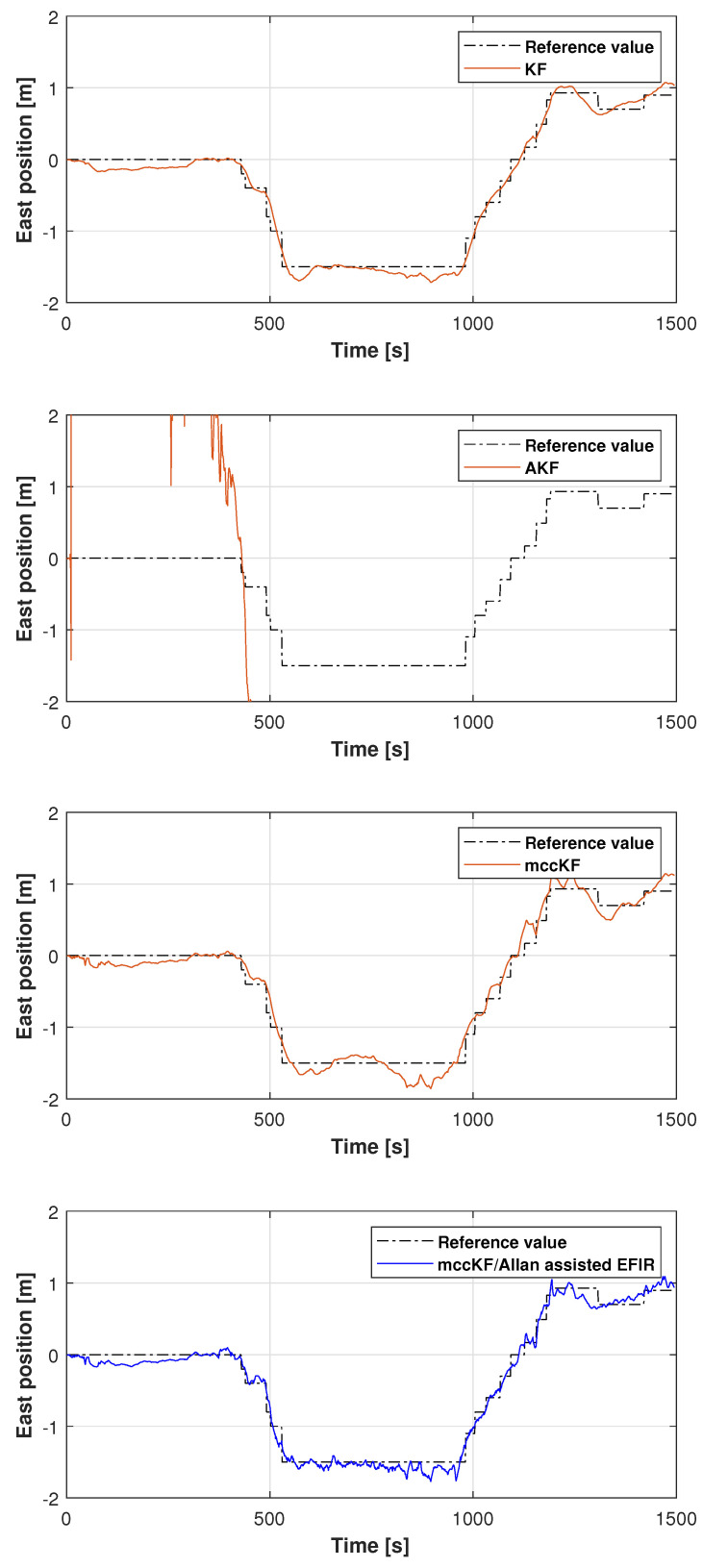
East position measured by the KF, AKF, mccKF, and mccKF/Allan-assisted FIR integrated filter in Test 2.

**Figure 13:**
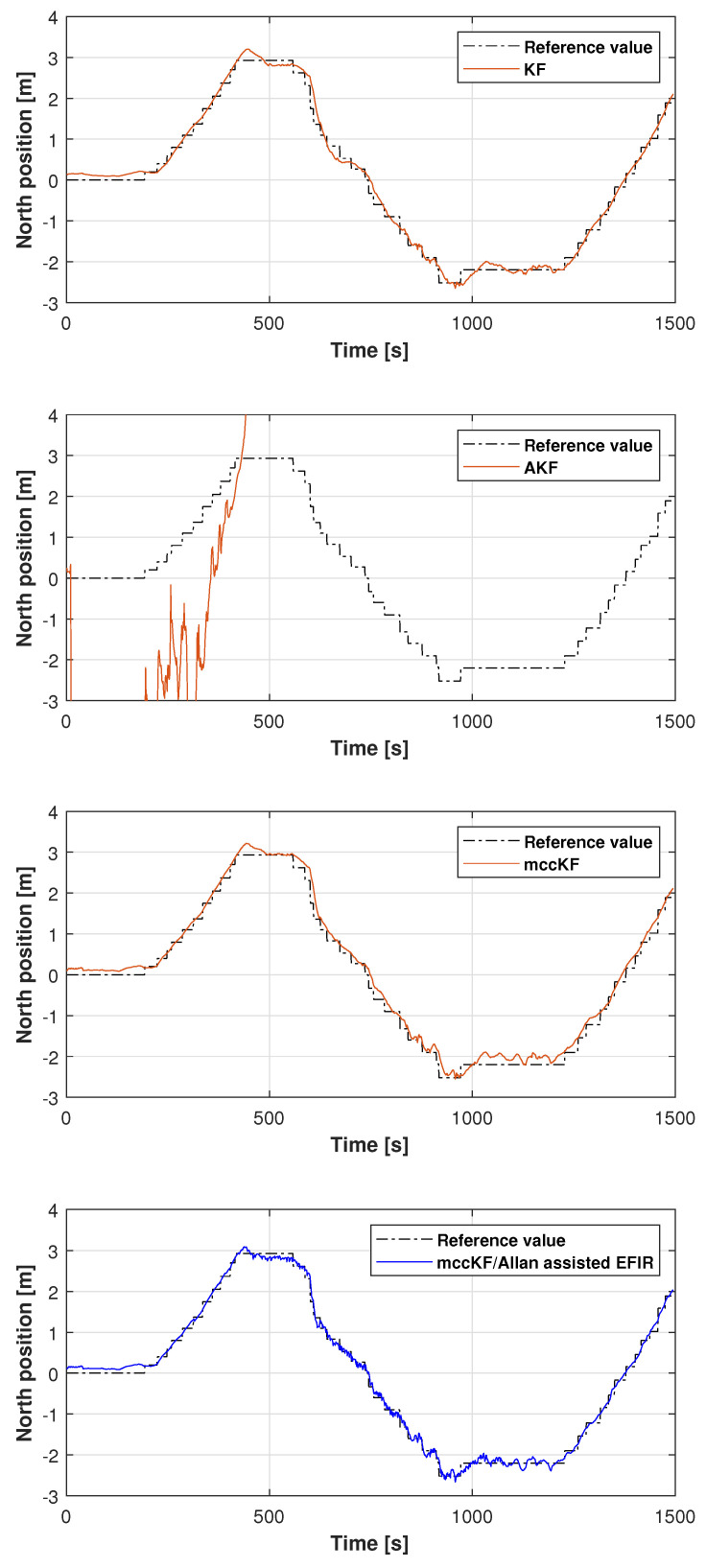
North position measured by the KF, AKF, mccKF, and mccKF/Allan-assisted FIR integrated filter in Test 2.

**Figure 14:**
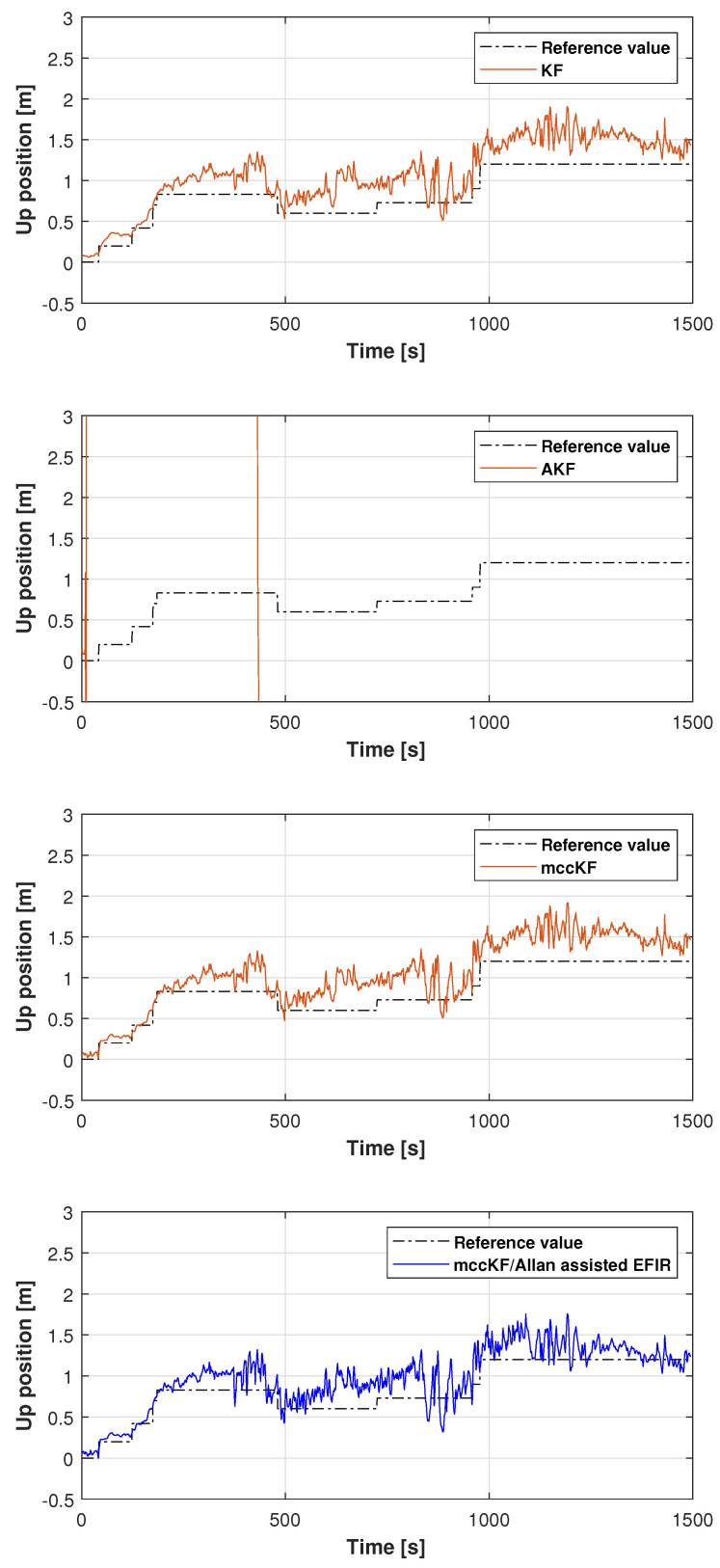
Upward position measured by the KF, AKF, mccKF, and mccKF/Allan-assisted FIR integrated filter in Test 2.

In this study, the real test was performed following another reference path, which is shown in Figure 11. Figure 12, Figure 13 and Figure 14 display the positions measured by the KF, AKF, mccKF, and the mccKF/Allan-assisted FIR integrated filter in the east, north, and upward directions in Test 2. The figures show that the performances of the filters in the east and north directions are different from those in the upward direction. In Figure 12 and Figure 13, similar to Test 1, the KF and mccKF exhibit some divergence in the east and north directions, particularly when the position changes. When compared with the KF and mccKF, the position of the proposed mccKF/Allan-assisted FIR filter is more stable, and its solution is closer to the reference value. In the case of upward direction, the positions of all the filters are similar. In Test 2, the estimation of the position in the upward direction by all the filters is initially very accurate, and later, a small amplitude of vibration is observed. Overall, the estimations of the target aircraft position by all the filters are achieved in this direction. For the filter, we set the following:

(41) $$Q_{k}=\left[\begin{array}{cc} Q_{1,k} & 0_{4\times 3}\\ 0_{2\times 4} & Q_{2,k}\\ 0_{1\times 4} & Q_{3,k} \end{array}\right]\times 0.1^{2}\;,$$

(42) $$Q_{1,k}=0.01\times \left[\begin{array}{cccc} 0.25\times \Delta k^{2} & 0.5\times \Delta k & 0 & 0\\ 0.5\times \Delta k & 1 & 0 & 0\\ 0 & 0 & 0.25\times \Delta k^{2} & 0.5\times \Delta k\\ 0 & 0 & 0.5\times \Delta k & 1 \end{array}\right]\;,$$

(43) $$Q_{2,k}=\left[\begin{array}{cc} 0.25\times \Delta k^{2} & 0.5\times \Delta k\\ 0.5\times \Delta k & 1 \end{array}\right]\times 0.05^{2}\;,$$

(44) $$R_{k}=\left[\begin{array}{cccc} 0.15 & 0 & 0 & 0\\ 0 & 0.15 & 0 & 0\\ 0 & 0 & 0.15 & 0\\ 0 & 0 & 0 & 0.01 \end{array}\right]\;,$$

(45) $$Q_{3,k}=1\;,$$

**Figure 15:**
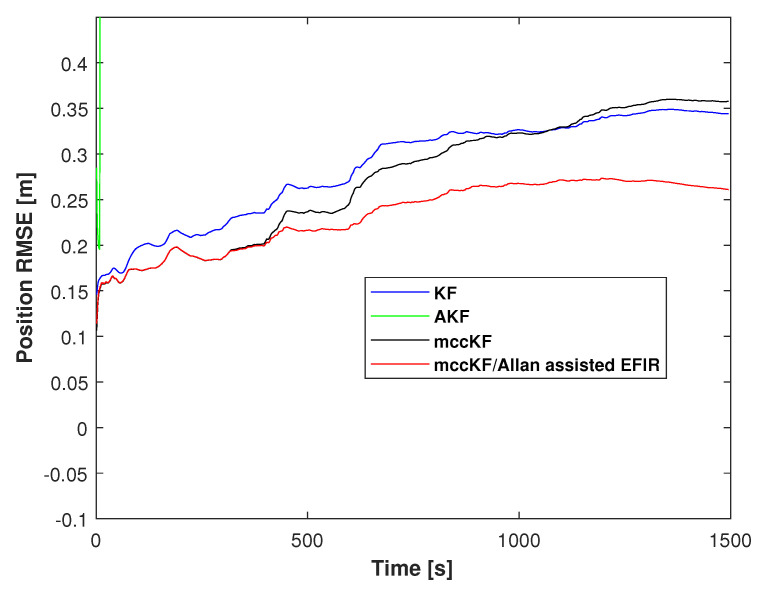
Position RMSEs of the KF, AKF, mccKF, and mccKF/Allan-assisted FIR integrated filter in Test 2.

**Figure 16:**
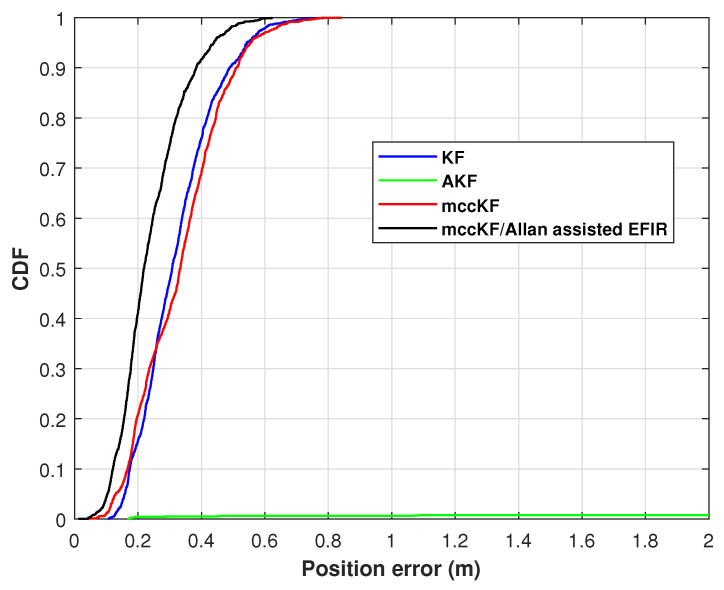
Position CDF of the KF, AKF, mccKF, and mccKF/Allan-assisted FIR integrated filter in Test 2.

The position RMSEs of the KF, mccKF, and mccKF/Allan-assisted FIR integrated filter in Test 2 are presented in Figure 15. The figure shows that the RMSEs of the mccKF and mccKF/Allan-assisted FIR are the same when the time index is <480 s. After 480 s, all the filters exhibit large differences. The localization error of the KF method rapidly increases, and this method has the largest RMSE when compared with the mccKF and proposed method. The increase in the RMSE of the mccKF is low compared with that of the KF. Compared with the KF and mccKF, although the RMSE of the proposed mccKF/Allan-assisted FIR integrated filter increases, it has the lowest value in all the filters. Table 2 lists the position RMSEs of the KF, mccKF, and mccKF/Allan-assisted FIR integrated filter in Test 2. The table reveals that, in comparison with the KF and mccKF, the proposed method exhibits the lowest RMSE. It reduces the localization error from the KF’s 0.18 m to 0.13 m, resulting in a reduction in the localization error by approximately 30%. The position CDFs of the KF, mccKF, and mccKF/Allan-assisted FIR integrated filter in Test 2 are shown in Figure 16. From the figure, we can see that the proposed method has the smallest localization error.

All the figures and the table indicate that, in Test 2, the proposed mccKF/Allan-assisted FIR filter is more efficient in minimizing localization errors when compared with the KF and mccKF. Tests 1 and 2 reveal that the proposed approach exhibits minimal localization error. Therefore, the suggested approach is the most effective.

## 5. Conclusions

To improve the robustness of the data fusion for INS-based integrated localization, an mccKF/FIR integrated filter was proposed herein. In this approach, the mccKF serves as the primary data fusion filter and is used for fusing data from multiple sources, including the INS. In this work, we used the *Mahalanobis* distance to verify the performance of the mccKF. If the performance of the mccKF is poor, the FIR filter is used to replace the mccKF. Two practical tests were performed to assess the performance of the suggested approach. The findings indicate that the mccKF/FIR integrated method is effective in minimizing localization errors when compared with the KF and mccKF. This observation emphasizes the efficacy of the proposed method.

## Figures and Tables

**Table 1:** Position RMSEs of the KF, mccKF, and mccKF/Allan-assisted FIR integrated filter in Test 1.

Algorithms	RMSE (m)			
	**East Direction**	**North Direction**	**Upward Direction**	**Mean**
KF	0.20	0.20	0.28	0.23
mccKF	0.15	0.14	0.28	0.19
mccKF/FIR	0.12	0.11	0.26	0.16

**Table 2:** Position RMSEs of the KF, mccKF, and mccKF/Allan-assisted FIR integrated filter in Test 2.

Algorithms	RMSE (m)			
	**East Direction**	**North Direction**	**Upward Direction**	**Mean**
KF	0.11	0.15	0.29	0.18
mccKF	0.14	0.17	0.27	0.19
mccKF/Allan-assisted FIR	0.10	0.10	0.20	0.13

## Data Availability

Data are contained within the article.

## Abbreviations

This manuscript employs the following abbreviations:

INS	Inertial Navigation System
KF	Kalman Filter
UWB	Ultra-Wideband
3D-SLAM	3D Simultaneous Localization and Mapping
UAVs	Unmanned Aerial Vehicles
PD	Parkinson’s Disease
ZUPT	Zero-velocity UPdaTe
MCC	Maximum Correntropy Criterion
GNSS	Global Navigation Satellite System
FIR	Finite Impulse Response
mccKF	Maximum Correntropy Criterion KF
LiDAR	Light Detection And Ranging
RTK	Real-Time Kinematics
RMSE	Root Mean Square Error
OD	Odometer
NHC	Nonholonomic Constraint
DVL	Doppler Velocity Log
MEMS	Microelectormechanical System
GPS	Global Positioning System
DRKF	Dual-Rate Kalman Filter
CKF	Cubature Kalman Filter
LWLR	Locally Weighted Linear Regression
LSTM	Long Short-Term Memory
CNS	Celestial Navigation System
EFIR	Extended Finite Impulse Response

## References

[1] Xu Y., Wan D., Shmaliy Y.S., Chen X., Tao S., Bi S. (2023). Dual Free-Size LS-SVM Assisted Maximum Correntropy Kalman Filtering for Seamless INS-Based Integrated Drone Localization. IEEE Trans. Ind. Electron..

[2] Chiang K.W., Tsai G.J., Li Y.H., Li Y., El-Sheimy N. (2020). Navigation Engine Design for Automated Driving Using INS/GNSS/3D LiDAR-SLAM and Integrity Assessment. Remote Sens..

[3] LaForest L., Hasheminasab S.M., Zhou T., Flatt J.E., Habib A. (2019). New Strategies for Time Delay Estimation during System Calibration for UAV-Based GNSS/INS-Assisted Imaging Systems. Remote Sens..

[4] Liu R., Wang Z., Qiu S., Zhao H., Wang C., Shi X., Lin F. (2022). A Wearable Gait Analysis and Recognition Method for Parkinson’s Disease Based on Error State Kalman Filter. IEEE J. Biomed. Health Inform..

[5] El-Sheimy N., Youssef A. (2020). Inertial sensors technologies for navigation applications: State of the art and future trends. Satell. Navig..

[6] Xu Y., Wang K., Jiang C., Li Z., Yang C., Liu D., Zhang H. (2023). Motion-Constrained GNSS/INS Integrated Navigation Method Based on BP Neural Network. Remote Sens..

[7] Wu J., Jiang J., Zhang C., Li Y., Yan P., Meng X. (2023). A Novel Optimal Robust Adaptive Scheme for Accurate GNSS RTK/INS Tightly Coupled Integration in Urban Environments. Remote Sens..

[8] Xu Y., Wang K., Yang C., Li Z., Zhou F., Liu D. (2023). GNSS/INS/OD/NHC Adaptive Integrated Navigation Method Considering the Vehicle Motion State. IEEE Sens. J..

[9] Qin X., Zhang R., Wang G., Long C., Hu M. (2023). Robust Interactive Multimodel INS/DVL Intergrated Navigation System With Adaptive Model Set. IEEE Sens. J..

[10] Fan Q., Sun B., Sun Y., Zhuang X. (2017). Performance Enhancement of MEMS-Based INS/UWB Integration for Indoor Navigation Applications. IEEE Sens. J..

[11] Zhao S., Huang B. (2020). Trial-and-error or avoiding a guess? Initialization of the Kalman filter. Automatica.

[12] Zhao L., Qiu H., Feng Y. (2015). Analysis of a Robust Kalman filter in loosely coupled GPS/INS navigation system. Measurement.

[13] Han S., Wang J. (2012). Integrated GPS/INS navigation system with dual-rate Kalman Filter. GPS Solut..

[14] Zhou W., Hou J., Liu L., Sun T., Liu J. (2017). Design and Simulation of the Integrated Navigation System based on Extended Kalman Filter. Open Phys..

[15] Feng K., Li J., Zhang X., Zhang X., Shen C., Cao H., Yang Y., Liu J. (2018). An Improved Strong Tracking Cubature Kalman Filter for GPS/INS Integrated Navigation Systems. Sensors.

[16] Liu D., Chen X. (2022). An ANN-Based Data Fusion Algorithm for INS/CNS Integrated Navigation System. IEEE Sens. J..

[17] Shmaliy Y.S. (2010). Linear optimal FIR estimation of discrete time-invariant state-space models. IEEE Trans. Signal Process..

[18] Xu Y., Shmaliy Y.S., Ahn C.K., Chen X., Guo H., Zhuang Y. (2021). Blind Robust Multi-Horizon EFIR Filter for Tightly Integrating INS and UWB. IEEE Sens. J..

[19] Zhao S., Shmaliy Y.S., Ahn C.K., Liu F. (2020). Self-tuning unbiased finite impulse response filtering algorithm for processes with unknown measurement noise covariance. IEEE Trans. Control Syst. Technol..

